# Incidence and Trend of Childhood and Adolescent Cancers in Yazd, Iran

**Published:** 2016-03-15

**Authors:** F Binesh, A Hashemi, M VakilI, MM Shakeri, R Masoumi Dehshiri

**Affiliations:** 1 **Department Of Pathology,Shahid Sadoughi University Of Medical Sciences,Yazd,Iran.**; 2 **Hematology and Oncology Research Center, Shahid Sadoughi University of Medical Sciences and Health Services,Yazd, Iran.**; 3 **Monitoring of Health Research Center, School of Medicine, Shahid Sadoughi University of Medical Sciences, Yazd, Iran. **; 4 **Shahid Sadoughi University of Medical Sciences, Yazd, Iran. **; 5 **Nutrition and Food Security Research Centre, Shahid Sadoughi University of Medical Sciences, Yazd, Iran.**

**Keywords:** Adolescent, Cancer, Childhood, Incidenc

## Abstract

**Background:**

Data on childhood and adolescent malignant tumors incidence are skimp in developing countries. In this study, we analyzed the incidence and trend of childhood and adolescent cancers in Yazd city, center of Iran between Jan 2004 and Dec 2013.

**Material and Methods:**

The various types of malignant tumors were grouped pursuant to the International Classification for Cancer in Children. To analyze the data, descriptive and illative statistical methods were used.

**Results:**

Two hundred twenty-two patients with a malignancy aged less than 18 years were studied with a male to female ratio of 1.36.The mean age of patients was 9.88 (±5.7) years. Leukemia with the frequency of 84 (37.8%) and after that lymphoid malignancies with the frequency of 49 (22.1%) were the most common cancers.

There was a low range of oscillation in the incidence rate of malignancies during this period of time (P value= 0.081). Malignancies were mostly in males (P value=0.057) but the whole process of malignancy incidence had gone toward the higher rate of incidence in females. Incidence rate of cancers types was steady. Malignancy incidence was 3-7 cases in hundred of thousands except a year of which this incidence rate was estimated 13.4.

**Conclusion:**

Leukemias and lymphomas were the main cancers in the center of Iran. Childhood and adolescent malignancies may be considerably under-recorded in our province .A childhood and adolescent cancer registry is necessary for exact analysis of these types of malignancies.

## Introduction

Cancer is the second prevalent cause of death among children in developed countries (1). Data on malignant tumor incidence are scanty in developing countries. Cancer in childhood has a very unpleasant effect on families and is a growing concern for public health, health systems, and society (2). Besides death, negative effect complications related to cancer issues remain in a great number of children for a long time which are brought on by cancer itself and its treatment (3). Moreover, evidences show that these factors have persistent negative effect on parents (4). Lack of knowledge about children’s cancer is one of the reasons for limitations in performing preventive interventions. Early detection and appropriate treatment are the key factors in survival of these patients (5). Studies show that cancer incidence among children has increased annually during the current three decades, whereas the mortality rate of it has decreased (6). Increased incidence of children’s cancer is also observed in European countries (7,8), America, and Canada (9,10,11). Possible reasons underlying the overall increase in childhood and adolescent cancer incidence fall into two major classes: (1) temporal changes in the prevalence of cancer risk factors and (2) increased cancer demonstration. The survival rate of these patients has also had an increasing process in such a way that one study of the American Cancer Society announced the 5-year survival rate of these patients to have been less than 50% in 1970 and about 80% at the present time (12). Moreover, other studies announced this rate to be about 81% in Europe (13). One recent study showed that cancer mortality for children and adolescents declined from 2000 to 2010, with considerable reduction ascertained for different cancer types (14). The context reasons for the observed worldwide progress in survival trends may enclasp the performance and execution of new chemotherapy protocols, multidisciplinary care involving oncologists, radiation oncologists, surgeons, pathologists, and radiologists and enlistment of patients in clinical trials. The context of cancers incidence and their trend pattern among children will help us to be able to use it in required predictions in order to represent diagnostic and treatment services. In addition, this information prepares input for subsequent investigation to improve quality of surveillance in these patients. Therefore, the authors decided to investigate the trend and incidence process of malignant tumors in patients aged less than 18 years in Yazd, Iran. 

## Material and Methods

The current research was a descriptive cross-sectional study. The target populations were the whole patients aged less than 18 years, who referred to Yazd Shahid Sadoughi Hospital, underwent sampling from the beginning of 2004 until the end of 2013 and was treated with the diagnosis of malignant tumor. Sampling was conducted in the form of census among the available records during this period of time. In this study, researcher-based checklist was used based on the previous studies and types of cancers were grouped according to the International Classification of Childhood Cancer .Data were collected from available records in archives of the department of pathology in Yazd Shahid Sadoughi Hospital during 10 years. All tumors were diagnosed on hematoxylin and eosin stained sections; and immunohistochemistry was applied wherever necessary. Fine-needle aspiration was done in some cases only. Bone marrow aspiration diagnoses in the case of leukemia were performed. Cytogenetic and molecular studies were not done. Patients who had tumors that were not classified by the ICCC-3 or who had in situ cancers were excluded from the final analysis. The patients whose record information was incomplete or the patients were not accessible to complete their records were also excluded. To analyze the data, descriptive and inferential statistical methods using SPSS 16 software were used.

## Results

**Figure 1:**
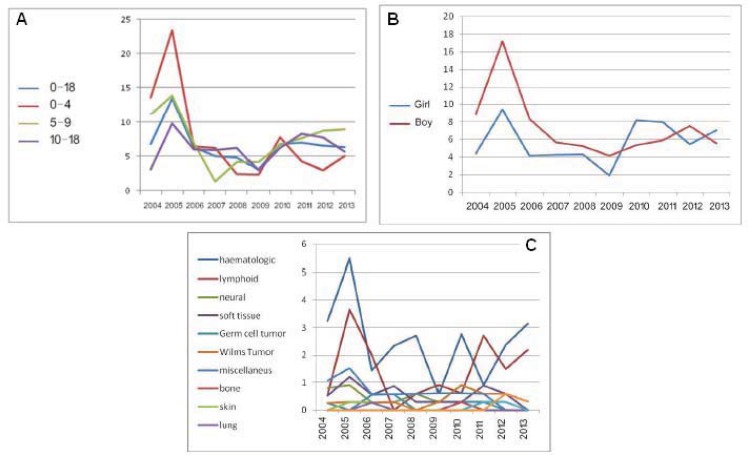
Total process of cancer incidence according to age (A), sex (B) and malignancy types (C).

In this research, 222 patients with malignancy aged less than 18 years were studied. The mean age of patients was 9.88 (±5.7) years and the range of 3 days-18years. One hundred twenty-eight of the patients (57.7%) were males and 94 patients (42.3%) were females. In the table incidenc of malignancies was seen. Leukemia incidence was 84(37.8%) and after that lymphoid malignancies with the frequency of 49 (22.1%) and these two cases totally constitute 60% of whole samples Chart 1 and table 2 visualize the total process of cancer incidence in age groups. The noticeable point which is demonstrated in this chart is the low range of oscillation in the incidence rate of malignancies in recent years that remained without any tremendous increase or decrease except in the chart 3, the incidence process of malignancy types is separately shown in which relatively steady incidence rate of cancer types can be mentioned. In this chart, the considerable incidence of hematologic and lymphoid malignancies compared with other malignancies is of great importance. Leukemia incidence was 3-7 cases in hundreds of thousands except a year of which this incidence rate was estimated 13.4. In second year of the study (P value= 0.081). In an investigation considering sex, it was seen that during the years of study, malignancies were mostly in males in a way that 128 cases (57.7%) of the total malignancies were in males and 94 of them (42.3%) were found in females (P value=0.057) (Figure 1B). But the whole process of malignancy incidence had gone toward the higher rate of incidence in females and this is of great importance.

**TableI:** *frequency of malignancy types in the study*

Type of **M**alignancy	Frequency	Percent
Hematologic malignancies	84	37.8%
Lymphoid malignancies	49	22.1%
CNS malignancies	19	8.6%
Soft tissue sarcoma	19	8.6%
Germ cell tumor	7	3.2%
Wilms tumor	13	5.9%
Miscellaneous	21	9.5%
Bone	2	0.9%
Skin	2	0.9%
Lung	1	0.5%
Thyroid	2	0.9%
Female genital tract	3	1.4%
Total	222	100

**TableII:** *incidence of malignancies according to age groups and studied years*

**Year**	**Population under 18 years**	**Age Groups**			**Total**
		10-18	5-9	0-4	
2004		3.1	13.08	13.62	6.73
2005		9.75	13.86	23.44	13.41
2006		5.97	7.01	6.47	66.3
2007		5.82	1.38	6.24	4.98
2008		6.19	4.16	2.39	4.78
2009		2.94	4.17	2.31	3.04
2010		6.19	6.63	7.76	6.72
2011		8.18	7.63	4.21	6.92
2012		7.75	8.65	2.98	6.54
2013		5.65	8.94	5.07	6.28

## Discussion

Overall, the incidence of cancers is still increasing especially in the less developed countries (15,16) but for some malignancies, the rates are really declining owing to progress in standard of living and intervention such as screening programs(17). More than 10% of all deaths in children below 15-years of age are caused by cancers in the developed communities. In the developing countries, childhood cancers are yet to be accepted as a main pediatric disease; however, they are fast rising as a distinguished entity to be dealt with (18). As a result, this matter has caused many social anxieties (19). In this study, the results showed that hematologic malignancies with 37.8% and lymphoid malignancies with 22.1% and after that nervous system malignancies and soft tissue sarcoma each with 8.6% had the highest rates in total malignancies. The general pattern of childhood and adolescent tumors in the present series seems to resemble the distribution of childhood and adolescent cancers reported in a study which was performed in Taiwan (20) and another study from Mexico(21). In a study by Ghasemi et al. on cancer pattern in children who referred to Bu Ali Sina Hospital in Sari (Mazandaran) from 2001-2010, indicated that among 148 patients with cancer aged 0-14, the most prevalent malignancy was Leukemia (30.5%), and then it was lymphoma (16.4%), melanoma (14.5%), and soft tissue sarcoma (9.5%)(22). However, in another study a higher incidence of CNS tumors, malignant bone tumors, and soft tissue sarcomas were reported (23). In a published research by Nabiha Missaoui et al., cancer incidence in children in Tunisia from 1993-2006 was studied and 727 patients were recorded. Considering the prevalence, leukemia with 27% was the most prevalent and then there was lymphoma with 25.7%, nervous system tumors with 9.2%, neuroblastoma with 7.7%, sarcomas with 6.9%, carcinomas with 6.3%, and bone tumors with 5.8 % respectively (24). It is totally shown that leukemia, lymphoma, and CNS malignancies are in all the most prevalent malignancies and this matter has been observed and emphasized in most of the studies. This incidence pattern is also seen in North America, South America, and Europe ,and it is only in Africa that lymphoma is of the highest rate of incidence (8,25) . In other words unlike many countries where leukaemias are the commonest childhood and adolescent tumors, in Africa they are much less common than lymphomas, especially Burkitt's lymphoma (26). Brain tumours are the most common solid tumors in children and adolescents in developed countries (27). They are not commonly reported in developing countries. To explain this it should be noted that the pattern of prevalence of pediatric malignancies has similarities, but there are considerable differences which highlights the importance of regional epidemiological studies. These differences may be due to the environmental or genetic differences (28).In addition, biologic factors or unavailability of special diagnostic tools like CT scan and MRI in some countries are also important. In the current study, 128 of the patients (57.7%) were males and 94 patients (42.3%) were females. Male predominance is a prominent feature of many childhood and adolescent tumors. In another study , males predominated (55%) in a ratio ranging from 1.3 to 3.1, depending on the type of tumor(21).Giun-Yi et al. in a similar work showed that the male-to-female incidence rate ratio was 1.19(20). Genetic difference in immune function may be responsible for the increased incidence of some tumors in males (29). In the present study, the view was that the total incidence process of malignancies proceeded to a higher rate of incidence in girls which is of great importance. One study revealed that overall cancer incidence rates of adolescents and young adults decreased in males whereas they increased in females (30). P D Baade showed that incidence rates for girls consistently increased by 0.9% per year (3). Arora et al. examined childhood cancer in India, a research in which the incidence, survival, and mortality rates of patients were investigated. They asserted that the incidence rate of cancer in children has increased in the past 25 years and this increase was higher in girls than in boys (31). This is the case that the incidence rate in boys was totally higher than that in girls in the present study and this rate was also higher in boys in similar studies (32).There are many disagreements on the age of cancer incidence in children but in a research by Baade et al. who examined 6184 patients aged 0-14 from 1983-2006, concluded that ages of 0-4 was the time of increase in malignancy incidence. There was no noticeable increase in malignancy incidence in older ages up to the year 1996 and this process has declined from this year on (3). In another research, the highest age incidence rate was 51.9 between 0 and 4 years (21). In contrast, Ghasemi et al. asserted that the highest incidence was in ages of 5-9 (22). NabihaMissaou et al. set forth their view that the most prevalent time for cancer incidence was ages of 10-14 (24). In the present study, research on patients’ age at the time of diseases incidence showed no especial pattern in malignancy incidence in age groups. In this study, there was no particular pattern in incidence process of malignancy in children. Similarly in another study, no significant increase trend was observed over the 1991-2005 period (33). Bravo et al. found no especial pattern in the process of this incidence as well (32). This process is under discussion in other studies so that Kaatsch et al. pointed out that this process is increasing in such a way that the annual growth in this area is 1.1% in Europe (34). It should be mentioned that increases in recorded childhood and adolescent malignancies incidence are widely reported in the developed world since the 1970s, increasing annually by 0.6% per year from 1975 to 2002 in the United States (9), by 1.0% in Europe between 1970 and 1999 (35) and by 1.0% in Sweden between 1960 and 1998(36). In a study in Taiwan during the 15-year study period, the incidence rates increased by 1% annually (20). Changes in trends could also be due to improvements in diagnostic procedures or registration programs (37). However, Giun-Yi believes that although such improvements may play a role, they are marginally effective (20). Furthermore, it is known that ethnic difference exists in the incidence of childhood and adolescent cancer (38). Innumerable risk factors and genetic factors are not well understood but may contribute to the observed changes .Exposure to some supposed risk factors such as electromagnetic fields or unusual infectious agents might have become more widespread over time. Malignancy incidence in children in the world differ between different countries The rate in the current study was 3 to 7 in hundred of thousands except a year in which this rate was 13.4. The incidence rate in a study done in Mexico was 15.03/ hundred of thousands /year (2010) for children of 0–18 years (21). The rate was 14.281 and 12.04/hundred of thousand/year for males and females, respectively in Taiwan (20). The previously published SEER data from the United States (39) has indicated an incidence of childhood cancer (0–19 years) of 16.6/ hundred of thousands person/year. It seems that childhood cancer incidence in Yazd is lower than that in all other countries .To explain this point we should mention that although this difference may be attributable to diversity in classification schemes and registration practices, an actual racial and geographical difference in predisposition to development of childhood and adolescent malignancies is suggested. Incidence rates of cancers vary between various racial groups within a single country and between different countries with similar ethnic compound. Such differences may be the result of genetic tendency, early or delayed exposure to infectious agents, and other environmental factors. On the other hand, some studies have stated that the incidence of childhood malignancies in low-income countries is lower than in industrialized countries (40). Registration of childhood cancer requires identification of symptoms, rapid access to a pediatric cancer unit, a correct diagnosis, and a data management foundation. In low-income countries, where these services are lacking, some children with cancer may die before diagnosis and registration.

In conclusion, in our province childhood and adolescent cancer may be considerably under-recorded. These results may help to enhance the level of public discourse and may lay the groundwork for subsequent works. Our findings suggest the importance of further epidemiologic studies and correct registration practices. A childhood cancer registry with correct data registration is recommended.

## Conflict of interest

The Authors have no conflict of interest.
